# Product quality, network effects, and efficiency of network markets

**DOI:** 10.3389/fpsyg.2022.1001445

**Published:** 2022-10-24

**Authors:** Dan Zhao, Yan Song

**Affiliations:** ^1^School of Business, Henan University of Science and Technology, Luoyang, China; ^2^School of Economics and Trade, Shandong Management University, Jinan, China; ^3^Shandong Technology Innovation Center of Social Governance Intelligence, Jinan, China

**Keywords:** network effects, consumer preference, efficient market, product quality, compatibility, competition model

## Abstract

The objective of our study is to capture the roles of product quality and network effects in the success and efficiency of network markets under strategic settings that defined in terms of market share as a strategic factor and profit as a financial indicator. The research paper shows that the efficiency of network markets depends heavily on the phase adjustment of competition models and the balance of network effects and product quality among enterprises. the network market is always efficient in price competition, but not true in quantity competition when the network effect difference is sufficiently large or/and the quality difference is relatively small, then network effects may play a perverse role in market efficiency. The main findings reveal that network effects do not always enhance the role of quality in market efficiency and market growth. The research outcomes point to high quality enterprises’ attitude toward compatibility with enterprises with large network effects. This research paper also offers insights on government intervention to correct the distorted impacts of sufficiently large network effects on the efficiency of network markets.

## Introduction

Nokia, one of the largest cellphone producers in the world for over 14 years, was surpassed by Apple and Samsung, whose iOS and Google Android-based products, respectively, have a higher quality that embodies the features of more powerful functions, easier operability, and more fluency in the running of applications. This example suggests that new entrants have the opportunity to defeat any incumbents, even those as strong as Nokia, by introducing products that have a higher quality than do the existing products. The example also suggests that the higher quality producer obtains the largest market share or profit, which implies the existence of an efficient market. However, the opposite is also probably true. The use of the Dvorak keyboard, which was used to break world records for speed typing, was proven by US Navy experiments to be more efficient than the use of a QWERTY keyboard. The increased efficiency of using Dvorak could amortize the cost of retraining a group of typists within the first 10 days of their subsequent full-time employment, but Dvorak has never replaced the QWERTY keyboard, which is not as well designed, but has been widely used for more than 100 years. Just as David (1985, p. 336) said, “competition in the absence of perfect futures markets drove the industry prematurely into standardization on the wrong system-where decentralized decision making subsequently has sufficed to hold it.” Similar examples are the initial success of Internet Explorer (vs. Netscape Navigator) and VHS (vs. Betamax), which show us that network effects resulting from standardization could swamp the impacts of quality and lead to an inefficient market.

Definitely, we can wonder if network effects lead to a perverse or inefficient market, and whether product quality or network effects are responsible for the success of network markets. In fact, for a long time, many scholars have debated on which factors drive the success of network markets and wonder if a market with large network effects can be efficient. The existing literature on network markets can be categorized into two strands. One strand argues that network effects are important but play a perverse role, leading to an inefficient market (Farrell and Saloner, 1985; Katz and Shapiro, 1985, 1994; Church and Gandal, 1996; Molina Castillo et al., 2011; Gutiérrez, 2020). Farrell and Saloner (1985) argue that an inferior standard with large network effects dominates whilst excess inertia can occur when information is incomplete and consumers have identical preferences. Church and Gandal (1996) find that standardization resulting in network effects is the equilibrium outcome if consumers prefer a variety of software to higher hardware technologies in hardware-software markets and if the market is socially inefficient when the hardware incumbent can commit to the installed base. Even though higher quality alternatives may be available, network effects may still hinder the entry of higher quality products into markets in which network effects are important by locking in low-quality standards or technologies (Katz and Shapiro, 1985, 1994). The market where network effects are important is of characteristic that history matters (Arthur, 1989; Besen and Farrell, 1994; Krugman, 1994; Molina Castillo et al., 2011; Gupta et al., 2019). All of them think that since consumers want compatibility with the installed base, higher quality products that are introduced later may be unable to replace the lower-quality ones, but earlier standards often depend crucially on historical accidents. Farrell and Klemperer (2007) point out that the lock-in phenomenon of network effects regarding consumers does indeed exist. Gutiérrez (2020) shows that network effects can overtake the quality effect if the market size is relatively small. In other words, network effects in network markets leading to consumer inertia, lock-in, or path dependence may help a lower-quality product defeat a higher quality one if the lower-quality product is widely expected to do so, resulting in an inefficient market.

The other strand emphasizes quality as a key factor in driving the success of products in network markets. Some scholars hold that product quality plays a significant, positive role not only on market share (Jacobson and Aaker, 1987; Kordupleski et al., 1993; Hilbolling et al., 2021; Zhao et al., 2021), but also on innovation (Winter, 2012; Bourke and Roper, 2017), or return on the stock market (Aaker and Jacobson, 1994; Tellis and Johnson, 2007; Xiong et al., 2020). Liebowitz and Margolis (1999) cite several cases to argue that product quality, rather than network effects, is the principal driver of market position. Indeed, network effects do not protect market participants from competition. Mcintyre (2011a,b) suggests that a large installed base may not be the sole competitive advantage for the success of products in network industries, but that the ability to produce higher quality products than can rivals is also important.

Obviously, scholars disagree on the critical drivers of the success of the market and whether the domination of network effects over product quality leads to an inefficient market or not. In order to settle such disputes, Tellis et al. (2009) is the first to attempt an empirical study of historical data in 19 categories. Their results show that product quality has greater impacts on market share than do network effects, which means that network markets are particularly efficient. Many researchers have commented, made rejoinders and raised a variety of questions and implications about this compelling finding (Brown and Morgan, 2009; Ratchford, 2009; Reibstein, 2009; Rossi, 2009; Shugan, 2009; Hu and Mcloughlin, 2012; Kim et al., 2014; Hilbolling et al., 2021; Cheng and Chan, 2022). These researchers agree that product quality and network effects have important impacts on market share, but disagree about product quality being able to overcome the influence of network effects and lead to an inefficient market. They point out that the findings regarding product quality’s dominating network effects on market share may be biased upward or downward because of excluded variables, such as price (Hilbolling et al., 2021), brand (Ratchford, 2009), advertisement (Reibstein, 2009), and customer uncertainty (Cheng and Chan, 2022). Findings and conclusions of Tellis et al. (2009) lack of theoretical grounds on the causality, cannot explain such examples as the dominance of Coca Cola over Pepsi, even though there is no apparent difference in product quality between them. Some scholars are suspicious of whether quality can always dampen network effects on market share whilst supporting the idea that network effects can enhance the positive effects of quality (Rossi, 2009; Hu and Mcloughlin, 2012; Kim et al., 2014).

Is it true that product quality always dominates network effects in network markets? What is the relationship between product quality and network effects? Are network markets always efficient? Do other factors, such as the strategic behaviors of enterprises and competition structure, have significant impacts on market outcomes? Do firms with higher product quality and have the same attitude toward product compatibility with firms with larger network effects? In our paper, we try to answer these questions primarily through a simple game-theoretical analysis regarding competition models, price, quality, and compatibility that do not provide by the above-mentioned scholars.

This paper makes the following contributions: (1) The efficiency of network markets depends heavily on the phase adjustment of competition models and the balance of network effects and product quality among enterprises, which supported in part by industrial data and case study (Tellis et al., 2009; Molina Castillo et al., 2011; Winter, 2012; Gretz and Basuroy, 2013; Kim et al., 2014; Hilbolling et al., 2021).

(1a) Network effects cannot protect the incumbent from competition and the entrant with high quality achieves a higher market share or more profit in equilibrium than does the incumbent with larger network effects, which means that the network market is always efficient in equilibrium in price competition (e.g., Proposition 2 without commitment and Proposition 4 with commitment in Supplementary Appendix). The conclusion indicates clearly that product quality is the critical driver in the success and efficiency of network markets, which is consistent with empirical studies by Tellis et al. (2009) and Kim et al. (2014).

(1b) When the competition model changes from price competition to quantity competition, the outcomes of network markets in efficiency depends on the quality difference Δ*s* and difference over network effects Δβ. Above conclusions hold if the network effect difference is small or/and the quality difference is large (e.g., $\Delta \,\beta \leq \beta_{1}^{{}^{**}}$ measured by strategic-factor market share, and $\beta_{1}^{{}^{**}}<\Delta \,\beta \leq \beta_{2}^{{}^{**}}$ or $\Delta \,\beta >\beta_{2}^{{}^{**}}$ and Δ*s* > 0.6181measured by financial-factor profit, in Proposition 1). Once the exception happens (e.g., $\Delta \,\beta >\beta_{2}^{{}^{**}}$, and Δ*s*≤0.6181 in Proposition 1), network effects become a barrier to entrants with higher quality and the incumbent with larger network effects obtains a higher market share or more profit. This means that network effects should be the primary driver in the success of network products in quantity competition, which is consistent with the conventional thinking. Although there is a network effect leading to consumer inertia or path dependence, it is more likely that there is insufficient friction (Katz and Shapiro, 1986, 1992). As the quality difference becomes large or the network effect difference small, product quality again becomes the critical driver in the success and efficiency of network markets. This shows that the product quality is as critical as the network effect (Rossi, 2009; Hu and Mcloughlin, 2012; Gretz and Basuroy, 2013).

(2) In contrast to a firm’s being unable to commit to output levels before consumers make their purchase decisions (FEE arises), the commitment to output by the incumbent may create strategic entry barriers that help the incumbent to defer, and even, block the entry of a new firm with high quality (Church and Gandal, 1996; Bresnahan, 1999). So, it is not true that entry is not urgent. Once the network effect plays a perverse role in the market efficiency, government intervention as a visible hand should exert influence on the distorted market, because large network effects may discourage the incentive to improve the quality (as discussed in what follows). So, government intervention may be necessary for and effective in correcting the distortion.

(3) the degree of compatibility with other products plays the opposite impacts on Firm 1 with higher quality and Firm 2 with larger network effects (as discussed in the following section “What impacts does compatibility have on the market outcome?”). Firm 1 with higher quality having the strong willingness to be compatible with the products of larger network effects, but Firm 2 with large network effects having the lack of incentive to be compatible with other products is quite different from what is stated by Katz and Shapiro (1992), who show that a firm introducing new technology is always biased against compatibility.

In the next section, we develop a game model firstly that captures the roles of product quality and network effects in the efficiency of the market by market share as a strategic factor and profit as a financial factor. An efficient market should be one in which the best-quality product emerges with the largest market share. The perspective of market share is consistent with the standard definition of efficiency in economics. Meanwhile, the best-quality producer should obtain the most profit in an efficient market because of the higher utility and willingness-to-pay of the consumers. We have also developed scenarios in which one firm with large network effects as an incumbent is or is not able to commit to output levels whilst another firm with high quality as a new entrant competes under the Cournot (quantity) and Bertrand (price) competition structures. Finally, we conclude the paper with some discussions and point out further potential research areas. Proofs of some of the propositions are given in the Supplementary Appendix.

## Model descriptions

Consider an industry consisting of two firms that produce goods with network effects. Firm 1 produces a good of high quality *s*_*1*_whilst Firm 2 produces with low-quality *s*_*2*_; therefore, *s*_1_ > *s*_2_. Let *s*_1_ = 1, and *s*_2_ = *s*, where *s* ∈ (0,1). The parameter *s*captures the product quality difference. A larger*s*implies closer substitutability between the two products and implies more homogeneity. A smaller *s*indicates a larger quality difference and more heterogeneity.

If consumers with different preferences buy nothing, their utilities are zero. If they buy a good with quality *s*_*i*_ at most, the utility function is $U_{i}=\theta \,s_{i}+v_{i}\,\left(q_{i}^{e}\right)-p_{i}$, *i* = 1,2, where θ is the marginal utility regarding quality and reflects consumer preferences, assuming a uniform distribution [0, 1]. The density function is 1. The larger θ is, the higher is the quality preferred by the consumers. The term $v_{i}\,\left(q_{i}^{e}\right)$ signifies a sole consumer’s evaluation of product *i* or the incremental utility (added willingness to pay for the network value of the good) of an individual consumer regarding network effects, and is an increasing function of $q_{i}^{e}$, where $q_{i}^{e}$ is the consumers’ expectations of the size of the network. According to the Metcalfe Law, let $v_{i}\,\left(q_{i}^{e}\right)=\beta_{i}\,q_{i}^{e}$, *i* = 1,2, where β_*i*_ ∈ (0,1) denotes network intensity and reflects network effects or network externalities. The larger β_*i*_ is, the higher is the willingness-to-pay by the consumers. We assume that Firm 2 with a low-quality product enters the market before Firm 1 with a high quality product. So, Firm 2’s products have much larger network effects than do those of Firm 1, owing to the time lag of generating network effects, namely, β_1_ < β_2_. For simplicity, we let β_1_ = 0, and β_2_ = β, where β ∈ (0,1), then we can derive *U*_1_ = θ−*p*_1_ and $U_{2}=\theta \,s+\beta \,q_{2}^{e}-p_{2}$.

The marginal cost of production is independent of the quantity and quality produced. A good with network effects often requires a large, initial lump sum for its development; however, once the good succeeds, the marginal production cost nearly becomes zero. Without a loss in generality, we can assume a zero-marginal cost. As long as the fixed costs are smaller than the firm’s equilibrium payoffs, the fixed costs have no effect on the equilibrium. To simplify this exposition, we assume that the fixed costs of production are equal to zero.

The consumer’s indifference between buying the high quality good and the low-quality good has a taste parameter, ${\bar{\theta }}_{1}$ (let *U*_1_ = *U*_2_) such that:


(1) $${\bar{\theta }}_{1}=\frac{p_{1}-p_{2}+\beta \,q_{2}^{e}}{1-s}$$

All the consumers for whom $\theta \geq {\bar{\theta }}_{1}$ will buy the high quality good by Firm 1. The consumer indifference between buying the differentiated good and not buying it at all has the taste parameter ${\hat{\theta }}_{1}=\left(p_{2}-\beta \,q_{2}^{e}\right)/s$. For this consumer, the purchase of the good of low-quality will imply a zero-utility level. Those customers described by ${\hat{\theta }}_{1}\leq \theta <{\bar{\theta }}_{1}$ will buy the low-quality good by Firm 2 whilst those described by $\theta <{\hat{\theta }}_{1}$ will not buy at all.

Given ${\hat{\theta }}_{1}$ and ${\hat{\theta }}_{1}$, the quantities demanded for the high and low-quality firms are, respectively, given by:


(2a) $$q_{1}=\int_{{\bar{\text{θ}}}_{1}}^{1}d\theta =1-\frac{p_{1}-p_{2}+\text{β}q_{2}^{e}}{1-s}$$

(2b) $$\;q_{2}=\int_{{\hat{\text{θ}}}_{1}}^{{\bar{\text{θ}}}_{1}}d\theta =\frac{p_{1}-p_{2}+\text{β}q_{2}^{e}}{1-s}-\frac{p_{2}-\text{β}q_{2}^{e}}{s}$$

The inverse demands are:


(3a) $$p_{1}=1-q_{1}-s\,q_{2}$$

(3b) $$p_{2}=s+\beta \,q_{2}^{e}-s\,q_{1}-s\,q_{2}$$

The profit functions are:


(4a) $$\pi_{1}=\left(1-q_{1}-s\,q_{2}\right)\,q_{1}$$

(4b) $$\pi_{2}=\left(s+\beta \,q_{2}^{e}-s\,q_{1}-s\,q_{2}\right)\,q_{2}$$

## When firm 2 cannot make a commitment

We assume that Firm 2 is unable to commit to quantity. That is to say, the consumers’ expectations about the sizes of the networks are formed after Firm 2 has selected its output levels, namely, Fulfilled Expectation Equilibrium (FEE). In order to ensure that the results are robust, we analyze the status quo in equilibrium and examine the market efficiency from market share and profit under Cournot quantity competition and Bertrand price competition.

### The status quo under Cournot competition

Under Cournot competition, both firms make decisions on quantity simultaneously. By solving the FOCs for maximizing profits as described by Eq. (4): $\frac{\partial \pi_{1}^{C}}{\partial q_{1}^{C}}=0$,$\frac{\partial \pi_{2}^{C}}{\partial q_{2}^{C}}=0$ and imposing a FEE condition satisfying $q_{2}^{e}=q_{2}^{C}$, we obtain the quantities and prices in equilibrium (superscript *C* represents the status quo under Cournot competition):


(5) $$q_{1}^{C}=\frac{2\,s-s^{2}-\beta }{4\,s-s^{2}-2\,\beta },q_{2}^{C}=\frac{s}{4\,s-s^{2}-2\,\beta }$$

(6) $$p_{1}^{C}=q_{1}^{C},p_{2}^{C}=s\,q_{2}^{C}$$

From Eqs (5, 6), we obtain the corresponding profits for the two firms under Cournot competition:


(7) $$\pi_{1}^{C}=p_{1}^{C}\,q_{1}^{C}={\left(q_{1}^{C}\right)}^{2}=\frac{{\left(2\,s-s^{2}-\beta \right)}^{2}}{{\left(4\,s-s^{2}-2\,\beta \right)}^{2}}$$

(8) $$\pi_{2}^{C}=p_{2}^{C}\,q_{2}^{C}=s\,{\left(q_{2}^{C}\right)}^{2}=\frac{s^{3}}{{\left(4\,s-s^{2}-2\,\beta \right)}^{2}}$$

To ensure that $q_{1}^{C}>0$ and $q_{2}^{C}>0$, we need 4*s*−*s*^2^−2β > 0 and 2*s*−*s*^2^−β > 0. As every consumer for whom θ ∈ [0,1] buys one good at most, Eq. (5) needs to satisfy $q_{1}^{C}+q_{2}^{C}\leq 1$. By considering the above restrictive conditions, we show that if β≤β^**^ = *s* holds, then $q_{1}^{C}>0$, $q_{2}^{C}>0$ and $q_{1}^{C}+q_{2}^{C}\leq 1$. Now that both firms have positive outputs with different quality and network effects, respectively, is product quality or network effects the factor that is more crucial to market share/profit? In other words, does Firm 1 with its high quality good or Firm 2 with its large network effects achieve a higher market share/more profit?

**Proposition 1.** *The two firms compete in Cournot mode and have positive outputs* (Δβ ≤ β^**^).

**FIGURE 1:**
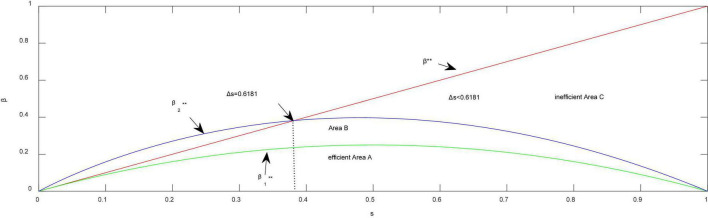
Changes of efficient boundaries in quantity competition. **Thresholds of network effects.

*Which firm achieves a higher market share or more profit depends on the quality difference*Δ*s and difference over network effects*Δβ:

(1) (*efficient Area A in* Figure 1) *when the network effect difference is small* ($0<\Delta \,\beta \leq \beta_{1}^{{}^{**}}=\left(1-s\right)\,s$), *for any*Δ*s* ∈ (0,1), *Firm 1 with high quality always achieves a higher market share* ($q_{1}^{C}\geq q_{2}^{C}$) *and more profit* ($\pi_{1}^{C}>\pi_{2}^{C}$).

(2) (*Area B in* Figure 1) *when the network effect difference is moderate* ($\beta_{1}^{{}^{**}}<\Delta \,\beta \leq \beta_{2}^{{}^{**}}=\left(2-s-\sqrt{s}\right)\,s$), *Firm 1 with high quality earns more profit* ($\pi_{1}^{C}\geq \pi_{2}^{C}$) *and Firm 2 with large network effects achieves a higher market share* ($q_{2}^{C}>q_{1}^{C}$).

(3) (*inefficient Area C in* Figure 1) *when the network effect difference is large* ($\Delta \,\beta >\beta_{2}^{{}^{**}}$), *and the quality difference between the two firms is relatively small* (Δ*s* ≤ 0.6181), *Firm 2 with large network effects achieves a higher market share* ($q_{2}^{C}>q_{1}^{C}$) *and earns more profit* ($\pi_{2}^{C}>\pi_{1}^{C}$);

**Proof:** See Supplementary Appendix B for the proof of Proposition 1.

From the standpoint of market share as a strategic factor, the reasoning behind Proposition 1 is that the absolute values of both product quality and network effects drive the success of the product in common, but the extent of driving the market is different, which crucially depends on the relative values of product quality and network effects between the two firms. Given the difference in product quality, if the difference over the network effects between the two firms is not sufficiently large ($\Delta \,\beta <\beta_{1}^{{}^{**}}$), it is unlikely to occur under the Cournot structure in which Firm 2 with larger network effects achieves higher market share. In that case, product quality has more impacts on the market than do network effects, which is consistent with the empirical findings (Tellis et al., 2009; Winter, 2012). However, if the difference over network effects between the two firms is sufficiently large ($\Delta \,\beta >\beta_{1}^{{}^{**}}$), network effects are the primary driver of success in the market supported by data collected from 255 innovative products (Gretz and Basuroy, 2013). Thus, Firm 2 with larger network effects acquires more market share than does Firm 1 with higher quality, which means that the market may be inefficient. In the American video game market, Nintendo has nearly 80% of the market share, although the sound and graphics of Nintendo’s 8-bit consoles are considered inferior to the 16-bit consoles provided by Sega (Shankar and Bayus, 2003).

From the standpoint of enterprise profit as a financial indicator, Proposition 1 also shows us that if the two firms compete in quantity and there is no leadership in quantity, Firm 1 with higher quality always earns more profit, provided that the quality difference between the two firms is sufficiently large (e.g., Δ*s* > 0.6181), whatever the difference may be over the network effects. However, if the difference between the two firms is not so large (e.g., Δ*s* ≤ 0.6181), Firm 2 with larger network effects will more likely dominate Firm 1 with higher quality, resulting in an inefficient market defining from both market share as a strategic factor and enterprise profit as a financial indicator, as long as the difference over the network effects is relatively large ($\Delta \,\beta >\beta_{2}^{{}^{**}}$).

### The status quo under Bertrand competition

Under Bertrand competition, both firms make decisions on price simultaneously. From Eq. (2), we obtain the two firms’ profit functions as follows:


(9) $$\pi_{1}^{B}=\left(1-\frac{p_{1}^{B}-p_{2}^{B}+\beta \,q_{2}^{e}}{1-s}\right)\,p_{1}^{B}$$

(10) $$\pi_{2}^{B}=\left(\frac{p_{1}^{B}-p_{2}^{B}+\beta \,q_{2}^{e}}{1-s}-\frac{p_{2}^{B}-\beta \,q_{2}^{e}}{s}\right)\,p_{2}^{B}$$

By solving the FOCs of maximizing profits described by Eqs (9, 10), the response functions can be described by:


(11) $$2\,p_{1}^{B}-p_{2}^{B}=1-s-\beta \,q_{2}^{e}$$

(12) $$s\,p_{1}^{B}-2\,p_{2}^{B}=-\beta \,q_{2}^{e}$$

By imposing the FEE condition and putting $q_{2}^{e}=q_{2}^{B}$ into Eq. (3), combined with Eqs (11, 12), we obtain the quantities and prices in equilibrium (superscript *B* represents the status quo under Bertrand competition):


(13) $$q_{1}^{B}=\frac{2\,s-2\,s^{2}-\beta }{\left(4\,s-s^{2}\right)\,\left(1-s\right)-\left(2-s\right)\,\beta },q_{2}^{B}=\frac{s\,\left(1-s\right)}{\left(4\,s-s^{2}\right)\,\left(1-s\right)-\left(2-s\right)\,\beta }$$

(14) $$p_{1}^{B}=\left(1-s\right)\,q_{1}^{B},p_{2}^{B}=s\,\left(1-s\right)\,q_{2}^{B}$$

From Eqs (13, 14), we obtain the corresponding profits for the two firms under Bertrand competition:


(15) $$\pi_{1}^{B}=p_{1}^{B}\,q_{1}^{B}=\left(1-s\right)\,{\left(q_{1}^{B}\right)}^{2}=\frac{\left(1-s\right)\,{\left(2\,s-2\,s^{2}-\beta \right)}^{2}}{{\left[\left(4\,s-s^{2}\right)\,\left(1-s\right)-\left(2-s\right)\,\beta \right]}^{2}}$$

(16) $$\;\;\pi_{2}^{B}=p_{2}^{B}\,q_{2}^{B}=s\,\left(1-s\right)\,{\left(q_{2}^{B}\right)}^{2}=\frac{s^{3}\,{\left(1-s\right)}^{3}}{{\left[\left(4\,s-s^{2}\right)\,\left(1-s\right)-\left(2-s\right)\,\beta \right]}^{2}}$$

To ensure that $q_{1}^{B}>0$ and $q_{2}^{B}>0$, we need (4*s*−*s*^2^)(1−*s*)−(2−*s*)β > 0 and 2*s*−2*s*^2^−β > 0. Since every consumer for whom θ ∈ [0,1] buys one good at most, Eq. (13) needs to satisfy $q_{1}^{B}+q_{2}^{B}\leq 1$. By considering the above restrictive conditions, we show that if $\beta \leq \beta^{{}^{*,**}}=\beta_{1}^{{}^{**}}=\left(1-s\right)\,s$ holds, then $q_{1}^{B}>0$, $q_{2}^{B}>0$ and $q_{1}^{B}+q_{2}^{B}\leq 1$. Now that both firms have positive outputs, product quality or network effects, which firm achieves a higher market share? Does higher market share also mean more profits?

**FIGURE 2:**
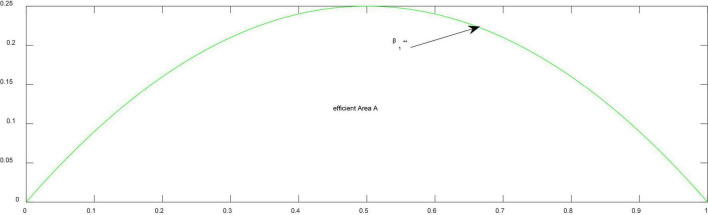
Changes of efficient boundaries in price competition. **Thresholds of network effects.

**Proposition 2.** (*efficient Area A in* Figure 2) *Two firms compete in Bertrand mode and have positive outputs* (Δβ ≤ β^*⁣**^). *Firm 1 with high quality always achieves no less market share and more profit than does Firm 2 with large network effects, whatever the quality and network effects. When both firms have the same market share, the market is covered*.

**Proof:** See Supplementary Appendix B for the proof of Proposition 2.

From Propositions 2, we find that Firm 1 with high quality always acquires no less market share, higher pricing and more profits than does Firm 2 if price competition in an industry is more likely to happen. This is to say that where price competition frequently occurs, an efficient market always emerges.

We can summarize Propositions 1 to 2 into the following Corollary 1.

**Corollary 1.** Firm 2 is unable to commit to output levels and both firms have positive quantities.

**FIGURE 3:**
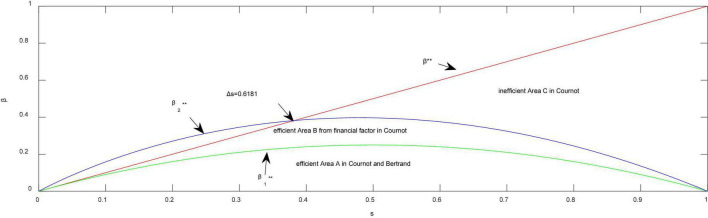
Changes of efficient boundaries both in Cournot and Bertrand. **Thresholds of network effects.

(1) *From the standpoint of strategy* (*efficient Area A in* Figure 3), *the market is always efficient in equilibrium when the two firms compete on price or the difference over network effects is sufficiently small* (*e.g.*,$\Delta \,\beta <\beta_{1}^{{}^{**}}$) *in quantity competition. Otherwise, a perverse market could emerge when the difference over network effects is sufficiently large* (*e.g.*,$\Delta \,\beta >\beta_{1}^{{}^{**}}$) *in quantity competition.*

(2) *From the standpoint of finance* (*efficient Area B in* Figure 3), *the market is always efficient, unless the difference over network effects is moderate* ($\beta_{1}^{{}^{**}}<\Delta \,\beta \leq \beta_{2}^{{}^{**}}$) *when the two firms compete on quantity.*

The reasoning behind Corollary 1 is that from the viewpoint of market share or profit, the market is likely to be inefficient for network markets unless price competition occurs frequently or the difference over network effects is sufficiently small in quantity structure. If the two firms compete in network markets with the aim of gaining the highest market share, the firm with high quality always obtains higher market share than does the firm with low-quality, regardless of the difference over network effects and of the presence of leadership. In our research, as the network effect difference decreases, there is no doubt that the firm with high quality gains greater market share or profits such that the firm surpasses other firms when the quality difference is relatively large. However, accompanied by the small quality difference and large network effect difference, the firm with large network effects will always gain higher market share or profits than do other firms. This statement is not opposed to the conventional thinking. In that case, network effects become the key driver of the success of network markets. Meanwhile, the market could still be perverse and inefficient.

As a matter of fact, we also wonder if the above findings are robust when the firm can commit outputs. In Supplementary Appendix A, we assume that Firm 2 can commit to announced output levels before consumers make their purchase decisions. We find that Corollary 2 is similar to Corollary 1. The significant difference lies in the difficulty of the entry of Firm 1 with high quality and the threshold of product quality or network effects that decide market share or profit. The ability to commit to output levels helps Firm 2 with large network effects to provide an increased barrier to entry in the market for Firm 1 with high quality. Once Firm 1 successfully enters the market where the network effect is important, it is more possible for Firm 2 with large network effects in the case that Firm 2 can commit itself to obtaining a higher market share or more profit than can Firm 1 with high quality, because the threshold of the quality or network effect difference is smaller than that of the case in which the firm cannot commit itself (e.g., $\beta_{2}^{C\,A\,C}<\beta_{2}^{{}^{**}}$).

According to Corollaries 1 and 2 (see Supplementary Appendix A), we obtain Theorem 1.

**Theorem 1.** *Regardless of whether a firm is able to commit to output levels or not, the network market is always efficient in equilibrium from either the viewpoint of market share or profit unless the quality difference is relatively small, but the difference over network effects is sufficiently large in quantity competition, and where network effects may play a perverse role in market efficiency.*

Once the role of network effects dominates, dampens the product quality, and becomes the primary key factor in driving the success of network markets, there is no doubt that the firm with a sufficiently large network effect will obtain higher market share or enterprise profit, especially when there is a small difference over product quality between the two firms. This is to say that the network effect may play a perverse role in network markets. For instance, although the iPhone for iOS by Apple was of higher quality than the smartphone Galaxy series for Android by Samsung, Apple still lost its first market share advantage all over the world, because of the sufficiently large difference over network effects. As the quality difference between the iPhone and Galaxy smartphones becomes even smaller, Apple may lose its position as the profit leader in smartphones to Samsung. Our suggestion is that when the gap in product quality inevitably becomes small, Apple should adjust its product prices in order to exert market power and correct market failure, as well as to increase more sales. It is a pity that the price of the new iPhone 5C released in 2013 was still expensive as if there were no price competition.

## Discussion

### Do network effects enhance the role of quality in a market?

On the one hand, some scholars hold the point of view that network effects enhance the positive effect of product quality on market efficiency and drive faster market growth (Economides and Himmelberg, 1995; Shapiro and Varian, 1999; Rohlfs, 2001; Tellis et al., 2009; Mcintyre, 2011b; Hu and Mcloughlin, 2012; Jung et al., 2019; Rietveld and Schilling, 2021). On the other hand, other researchers argue that network effects may have a substantial chilling or dampening effect on market growth and innovativeness (Choi, 1997; Rogers, 2003; Krafft and Salies, 2008; Goldenberg et al., 2010; Gretz and Basuroy, 2013; Bourke and Roper, 2017). As a matter of fact, network effects and product quality are not always mutually complementary in improving market efficiency. From the standpoint of market share, suppose that two firms compete in Cournot structure. We can easily obtain the output for Firm 2 by strictly increasing the network effect (e.g.,$\frac{\partial q_{2}^{C}}{\partial \beta }>0$), which has an inverted U-shaped relationship with its product quality (e.g., $\frac{\partial q_{2}^{C}}{\partial s}>0$ when β < *s*^2^/2 and $\frac{\partial q_{2}^{C}}{\partial s}<0$ when β > *s*^2^/2) from Eq. (5). This means that if the network effect is greater than or equal to 0.5, β > *s*^2^/2 holds for any*s* ∈ (0,1), so that $\frac{\partial q_{2}^{C}}{\partial s}<0$. The network effects do not motivate Firm 2 to improve quality. If the network effect is less than 0.5, the opposite case occurs. Therefore, whether the network effects enhance the role of quality in market efficiency and market growth depends crucially on the balance between the quality and the threshold of the network effects.

### To be better rather than to be the first?

According to Theorem 1, if two firms compete in price mode, which firm will win simply depends on product quality, which seems to be the only one of success, so path dependence or consumer inertia is not so important as they are in the conventional thinking. Thus, firms should put focus on quality rather than such factors as the timing in entering a market. However, it is not always true that it is better to be better than to be the first. According to Theorem 1, if two firms compete in quantity whilst the network effect is sufficiently large and whatever the quality difference, Firm 2 with large network effects defeats Firm 1 with high quality in market share or profit, showing that a first entry into the market that results in a large installed base and path dependence could be more important than the other factors. This statement is in accord with the first mover advantage (Golder and Tellis, 1993; Shankar et al., 1998; Tellis and Golder, 2001), so the timing of the entry is still urgent (Shugan, 2009; Wunker, 2012). Therefore, it should not go too far on the topics of whether it is better to be better or to be the first, and what strategies should be taken depending on the balance between the quality difference and network effect difference.

### What impacts does compatibility have on the market outcome?

Sections “When firm 2 cannot make a commitment” and “Discussion” show us the status quo when a high quality product is completely incompatible with a low-quality one. However, are there any changes in market share or profit due to the degree of compatibility with other products? For example, each Windows versions by Microsoft is backward compatible with older versions. An exception was the initial Vista version, which did not provide enough compatibility with the older XP version, as well as older programs, resulting in a market share that was lower than that of XP and surpassed by that of the subsequent Windows 7 version in 2012. We also provide some simple proofs to show the impacts of compatibility on market outcomes.

Suppose that two firms compete in Cournot quantity and no firm can commit to output levels (FEE) and that a high quality product is completely compatible with a low-quality product, then the two firms’ outputs are, respectively, by some trivial calculations:


(17) $$q_{1}=\frac{2\,s-s^{2}-\left(1-s\right)\,\beta }{4\,s-s^{2}-2\,\beta },q_{2}=\frac{s+\left(1-s\right)\,\beta }{4\,s-s^{2}-2\,\beta }$$

We can easily obtain if and only if β≤*s*/2, and *q*_1_ > 0, *q*_2_ > 0 and *q*_1_ + *q*_2_≤1 hold. From the viewpoint of market share, we find that *q*_1_−*q*_2_≥0 holds for any β ∈ (0,*s*/2]. This means that if a high quality product were completely compatible with a low-quality one, Firm 1 with high quality achieves the same or a greater market share than does Firm 2 with large network effects. In contrast to Proposition 1, the result shows that the market share of the high quality product increases whilst that of the low-quality product decreases with the degree of compatibility, thereby improving the market efficiency. So, it is a wise choice for the entrant with high quality to improve the degree of compatibility with other products, especially those with larger network effects.

## Conclusion

This paper addresses an ongoing debate on the critical driver for the success of a market in which the network effect is important. We constructed a game-theoretical model in response to the empirical study under the scenario that one firm with large network effects was an incumbent and another firm with a high quality product was a new entrant competing in a network industry. The analysis was conducted under price (Bertrand) and quantity (Cournot) competitions to determine whether a firm was able to commit to output levels or not to keep the conclusions complete, precise and robust. The results show that whether a firm is able to commit to output levels or not (whether there is a FEE or not), and from the viewpoint of market share or profit, when the two firms compete in price, network effects cannot protect the incumbent from competition and the entrant with high quality achieves a higher market share or more profit in equilibrium than does the incumbent with large network effects, which means that the network market is always efficient in equilibrium. The conclusion indicates clearly that product quality is the critical driver in the success and efficiency of network markets.

When the two firms compete in quantity, the above conclusion is true unless the quality difference is small or/and the network effect difference is large. Once the exception happens, the network effect becomes a barrier to entrants with high quality and the incumbent with large network effects obtains a higher market share or more profit. This means that the network effects should be the primary driver in the success of network products, which is consistent with the conventional thinking. Although there is a network effect leading to consumer inertia or path dependence, it is more likely that there is insufficient friction. As the quality difference becomes large or the network effect difference small, product quality again becomes the critical driver in the success and efficiency of network markets.

In contrast to a firm’s being unable to commit to output levels before consumers make their purchase decisions, the commitment to output by the incumbent may create strategic entry barriers that help the incumbent to defer, and even, block the entry of a new firm with high quality. So, it is not true that entry is not urgent. Once the network effect plays a perverse role in the market efficiency, government intervention as a visible hand should exert influence on the distorted market, because large network effects may discourage the incentive to improve the quality. So, government intervention may be necessary for and effective in correcting the distortion.

Finally, the degree of compatibility with other products plays the opposite impacts on Firm 1 with higher quality and Firm 2 with larger network effects, as discussed in section “What impacts does compatibility have on the market outcome?”. Firm 1 with higher quality having the strong willingness to be compatible with the products of larger network effects, but Firm 2 with larger network effects having the lack of incentive to be compatible with other products.

This study has several limitations that could be addressed by further research. We sketch a simple game model in a static way that captures the roles of quality and network effects in the success of network markets. As a matter of fact, it is likely to examine the roles by modeling the dynamics in an inter-temporal or evolutionary setting with industry data.

## Data availability statement

The original contributions presented in this study are included in the article/Supplementary material, further inquiries can be directed to the corresponding author.

## Author contributions

DZ: conceptualization, writing—review and editing, project administration, and funding acquisition. YS: writing—original draft, software, validation, methodology, supervision, resources, and formal analysis. Both authors contributed to the article and approved the submitted version.
