# Supplementary material for: Product quality, network effects, and efficiency of network markets
Source: Front Psychol. 2022 Oct 24;13:1001445. doi: 10.3389/fpsyg.2022.1001445 (PMC9637856; doi:10.3389/fpsyg.2022.1001445)
Supplement: Supplementary file 1 [file Data_Sheet_1.PDF]

# Appendix A

***When Firm 2 can make a commitment***

In this section, we assume that Firm 2 can commit to announced output levels before consumers make their purchase decisions. As in Section 3, we analyze the status quo in equilibrium for the market share and profit under Cournot quantity competition and Bertrand price competition.

*1. The status quo under Cournot competition*

Since Firm 2 can commit to announced output levels before consumers make their purchase decisions, the inverse demand functions for the two firms under Cournot Competition are rewritten as (superscript *CAC* represents the status quo under Cournot competition whilst Firm 2 is able to make a commitment):

$$\begin{aligned} p_1^{CAC} &= 1 - q_1^{CAC} - sq_2^{CAC} \\ p_2^{CAC} &= s - sq_1^{CAC} + (\beta - s)q_2^{CAC} \end{aligned} \tag{A1}$$

The profit functions are rewritten as:

$$\begin{aligned} \pi_1^{CAC} &= (1 - q_1^{CAC} - sq_2^{CAC})q_1^{CAC} \\ \pi_2^{CAC} &= [s - sq_1^{CAC} + (\beta - s)q_2^{CAC}]q_2^{CAC} \end{aligned} \tag{A2}$$

By solving the FOCs of maximizing profits as described by Eq. (A2), we obtain the quantities and prices in equilibrium:

$$q_1^{CAC} = \frac{2s - s^2 - 2\beta}{4s - s^2 - 4\beta}, q_2^{CAC} = \frac{s}{4s - s^2 - 4\beta} \tag{A3}$$

$$p_1^{CAC} = q_1^{CAC}, p_2^{CAC} = (s - \beta)q_2^{CAC} \tag{A4}$$

To ensure that $q_1^{CAC} > 0$ and $q_2^{CAC} > 0$, we need $4s - s^2 - 4\beta > 0$ and $2s - s^2 - 2\beta > 0$. Since every consumer for whom $\theta \in [0,1]$ buys one good at most, Eq. (A3) needs to satisfy $q_1^{CAC} + q_2^{CAC} \le 1$. By considering the above restrictive conditions, we show that if $\beta \le \beta^{CAC} = \frac{s}{2}$ holds, then $q_1^{CAC} > 0$, $q_2^{CAC} > 0$ and $q_1^{CAC} + q_2^{CAC} \le 1$.

**Proposition 3.** *Suppose that Firm 2 is able to commit to outputs and the two firms have positive outputs in Cournot mode ($\Delta\beta \le \beta^{CAC}$). Whatever the quality difference $\Delta s \in (0,1)$, which firm achieves a higher market share depends on the difference over network effects:*

*(1) If the network effect difference is small ($0 < \Delta\beta < \beta_1^{CAC} = \frac{(1-s)s}{2}$), Firm 1 with high quality achieves a higher market share ($q_1^{CAC} > q_2^{CAC}$);*

*(2) If the network effect difference is moderate ($\Delta\beta = \beta_1^{CAC}$), Firm 1 with high quality and Firm 2 with large network effects have the same market share ($q_1^{CAC} = q_2^{CAC}$);*

*(3) If the network effect difference is large ($\Delta\beta > \beta_1^{CAC}$), Firm 2 with large network effects achieves a higher market share ($q_2^{CAC} > q_1^{CAC}$).*

See Appendix B for the proof of Proposition 3.

From Eqs. (A3) and (A4), we obtain the corresponding profits for the two firms under Cournot competition:

$$\pi_1^{CAC} = \frac{(2s - s^2 - 2\beta)^2}{(4s - s^2 - 4\beta)^2} \tag{A5}$$

$$\pi_2^{CAC} = \frac{(s - \beta)s^2}{(4s - s^2 - 4\beta)^2} \tag{A6}$$

Does higher market share mean more profit?

**Proposition 4.** *Two firms compete in Cournot mode and have positive outputs ($\beta \le \beta^{**}$). When the quality difference between the two firms is relatively large ($\Delta s > 0.5$), whatever the network effects, Firm 1 with high quality obtains more profit ($\pi_1^{CAC} > \pi_2^{CAC}$). However, when the quality difference between the two firms is relatively small ($\Delta s \le 0.5$), which firm obtains more profit depends on the difference over network effects:*

*(1) If the network effect difference is small ($0 < \Delta\beta < \beta_2^{CAC} = (1-s)s$), Firm 1 with high quality obtains more profit ($\pi_1^{CAC} > \pi_2^{CAC}$);*

*(2) If the network effect difference is moderate ($\Delta\beta = \beta_2^{CAC}$), both Firm 1 with high quality and Firm 2 with large network effects have the same profit ($\pi_1^{CAC} = \pi_2^{CAC}$);*

*(3) If the network effect difference is large ($\Delta\beta > \beta_2^{CAC}$), Firm 2 with large network effects obtains more profit ($\pi_2^{CAC} > \pi_1^{CAC}$).*

See Appendix B for the proof of Proposition 4.

In contrast to Propositions 1 and 2, Propositions 3 and 4 show us that when Firm 2 can commit to output levels in advance, it is easier for Firm 2 with large network effects to deter Firm 1 with high quality from entering the market (e.g., $\beta^{CAC} < \beta^{**}$), and it is more likely for Firm 2 to achieve higher market share or more profit provided that the difference over network effects is large over the threshold (e.g., $\beta < \beta_1^{**}$). This means that Firm 1 has more difficulty in acquiring higher market share even if the quality difference between the two firms is large. An inefficient market is more likely to arise, because the threshold of the difference over the network effects is smaller than the status quo of FEE (e.g., $\beta_1^{CAC} < \beta_1^{**}$).

*2. The status quo under Bertrand competition*

Under Bertrand competition, both firms make decisions on price simultaneously. Since Firm 2 can commit to announced output levels before consumers make their purchase decisions, the corresponding profit functions for the two firms from Eqs. (2), (3) and (4) are rewritten as (superscript *BAC* represents the status quo under Bertrand competition whilst Firm 2 is able to make a commitment):

$$\pi_1^{BAC} = [1 - \frac{(\beta - s)p_1^{BAC} + sp_2^{BAC}}{\beta - s + s^2}]p_1^{BAC} \tag{A7}$$

$$\pi_2^{BAC} = (\frac{-sp_1^{BAC} + p_2^{BAC}}{\beta - s + s^2})p_2^{BAC} \tag{A8}$$

By solving the FOCs of maximizing profits described by Eqs. (A7) and (A8), the response functions can be described by:

$$2(\beta - s)p_1^{BAC} + sp_2^{BAC} = \beta - s + s^2 \tag{A9}$$

$$sp_1^{BAC} - 2p_2^{BAC} = 0 \tag{A10}$$

By some standard calculations, we obtain the quantities and prices in equilibrium:

$$q_1^{BAC} = \frac{2(s-\beta)}{4s - s^2 - 4\beta}, q_2^{BAC} = \frac{s}{4s - s^2 - 4\beta} \tag{A11}$$

$$p_1^{BAC} = \frac{2(s - s^2 - \beta)}{4s - s^2 - 4\beta}, p_2^{BAC} = \frac{s(s - s^2 - \beta)}{4s - s^2 - 4\beta} \tag{A12}$$

To ensure that Eqs. (A11) and (A12) are greater than zero, we must ensure that $4s - s^2 - 4\beta > 0$ and $s - s^2 - \beta > 0$ hold. Since every consumer for whom $\theta \in [0,1]$ buys one good at most, Eq. (A10) needs to satisfy $q_1^{BAC} + q_2^{BAC} \le 1$. By considering the above restrictive conditions, we show that if $\beta \le \beta^{BAC} = \frac{(1-s)s}{2}$ holds, then $q_1^{BAC} > 0$ , $q_2^{BAC} > 0$ and $q_1^{BAC} + q_2^{BAC} \le 1$. Now that both firms have positive outputs, product quality or network effects, which firm achieves higher market share?

**Proposition 5.** *Suppose that Firm 2 is able to commit to outputs and that the two firms have positive outputs in Bertrand mode ($\Delta\beta \le \beta^{BAC}$). Whatever the quality difference or the network effect difference between the two firms, Firm 1 with high quality always achieves a higher market share than does Firm 2 with high network effects ($q_1^{BAC} > q_2^{BAC}$).*

**Proof**: By Eq. (A11), we obtain $q_1^{BAC} - q_2^{BAC} = \frac{s - 2\beta}{4s - s^2 - 4\beta}$. Since $\frac{s}{2} > \frac{(1-s)s}{2} = \beta^{BAC}$ for any $s \in (0,1)$, then $q_1^{BAC} - q_2^{BAC} > 0$.

Firm 1 with high quality achieves a higher market share than does Firm 2 with large network effects. Does higher market share mean more profits?

**Proposition 6.** *Suppose that Firm 2 is able to commit to output and that the two firms have positive outputs in Bertrand mode ($\Delta\beta \le \beta^{BAC}$). Firm 1 with high quality achieves more market share and obtains more profit than does Firm 2 with large network effects for any $\Delta s \in (0,1)$.*

**Proof**: From Eqs. (A11) and (A12), we obtain $\pi_1^{BAC} - \pi_2^{BAC} = \frac{s(1-s) - \beta}{[(4s - s^2)(1-s) - (2-s)\beta]^2}$. As

$\beta \le \frac{s(1-s)}{2} = \beta^{BAC}$, then $\pi_1^{BAC} > \pi_2^{BAC}$ always holds for any $\Delta s \in (0,1)$.

Like the status quo under the Cournot structure, when Firm 2 can commit to output levels in advance and the two firms compete on price, there is increased difficulty in entering the network market for Firm 1 with high quality (e.g., $\beta^{BAC} < \beta^{***}$). Yet, the market outcome is different from the above structures because Firm 1 always dominates Firm 2 with large network effects upon market share, which means that the market is efficient as under Bertrand structure, in which Firm 2 could not commit itself once Firm 1 had entered successfully.

We can summarize Propositions 3–6 into the following Corollary 2.

**Corollary 2.** *Suppose that Firm 2 can make a commitment and both firms have positive outputs.*

*(1) From the standpoint of market share, the network market is always efficient when the two firms compete on price or the network effect difference is small ($\Delta\beta < \beta_1^{CAC}$) in quantity competition. Otherwise, the network market is inefficient if the difference over network effects is sufficiently large ($\Delta\beta > \beta_1^{CAC}$) in quantity competition.*

*(2) From the standpoint of profit, the network market is efficient when the two firms compete in price or the network effect difference is small ($\Delta\beta < \beta_2^{CAC}$) in quantity competition. Otherwise, network effects could lead to a perverse market; namely, the network market is inefficient if the quality difference is relatively small and the difference over network effects is sufficiently large ($\Delta s \le 0.5$ and $\Delta\beta > \beta_2^{CAC}$) under Cournot structure.*

# Appendix B

***Proof of Proposition 1***

By Eq. (5), we obtain $q_1^C - q_2^C = [(1-s)s - \beta]/(4s - s^2 - 2\beta)$. Since $\beta^{**} = s > (1-s)s = \beta_1^{**}$, $q_2^C > q_1^C$ for $\beta > \beta_1^{**}$, $q_1^C > q_2^C$ for $\beta < \beta_1^{**}$, and $q_1^C = q_2^C$ for $\beta = \beta_1^{**}$.

From Eqs. (7) and (8), we obtain $\pi_1^C - \pi_2^C = A_1/(4s - s^2 - 2\beta)^2$, where $A_1 = \beta^2 - 2s(2-s)\beta + (4 - 5s + s^2)s^2$. Obviously, Sign {$\pi_1^C - \pi_2^C$} = Sign {$A_1$}. $A_1$ is a quadratic curve about $\beta \in (0, \beta^{**}]$, and $\Delta_1 = 4s^3 > 0$. So, there are two roots for equation $A_1 = 0$. One root, $(2 - s + \sqrt{s})s > s = \beta^{**}$, always holds. If the other root, $\beta_2^{**} = (2 - s - \sqrt{s})s \le \beta^{**}$, holds, we must ensure that $s + \sqrt{s} - 1 \ge 0$. By some simple calculations, we know that $s + \sqrt{s} - 1 < 0$ for $s \in (0, \frac{3-\sqrt{5}}{2} \approx 0.3819]$, thus $\beta_2^{**} > \beta^{**}$ and $s + \sqrt{s} - 1 \ge 0$ for $s \in (0.3819,1)$, thus $\beta_2^{**} \le \beta^{**}$.

For $s \in (0,0.3819]$, quadratic curve $A_1$ goes upwards with two roots that are larger than $\beta^{**}$, so $\pi_1^C > \pi_2^C$ always holds for any $\beta \le \beta^{**}$, but for $s \in (0.3819,1)$, quadratic curve $A_1$ goes

upwards with one root that is smaller than $\beta^{**}$ and the other root is larger than $\beta^{**}$, so if $0 < \beta < \beta_2^{**}$, $\pi_1^C > \pi_2^C$ always holds. If $\beta \geq \beta_2^{**}$, $\pi_2^C \geq \pi_1^C$ always holds.

***Proof of Proposition 2***

From Eq. (13), we obtain $q_1^B - q_2^B = \dfrac{(1-s)s-\beta}{(4s-s^2)(1-s)-(2-s)\beta} \geq 0$ for any $\beta \leq \beta^{***}$.

From Eqs. (15) and (16), we obtain $\pi_1^B - \pi_2^B = A_2 / [(4s-s^2)(1-s)-(2-s)\beta]^2$, where $A_2 = \beta^2 - 4s(2-s)\beta + (4-s)(1-s)^2 s^2$. Obviously, Sign $\{\pi_1^B - \pi_2^B\}$ = Sign $\{A_2\}$. $A_2$ is a quadratic curve about $\beta \in (0, \beta^{***}]$, and $\Delta_2 = 4s^3(1-s)^2 > 0$. So, there are two roots about equation $A_2$=0 . Quadratic curve $A_2$ goes upwards with which both roots $(2 \pm \sqrt{s})(1-s)s > (1-s)s = \beta^{***}$ always hold, so $A_2$>0 always holds, and thus, $\pi_1^B > \pi_2^B$ for any $\beta \in (0, \beta^{***}]$.

***Proof of Proposition 3***

By Eq. (A3), we obtain $q_1^{CAC} - q_2^{CAC} = [(1-s)s-2\beta]/(4s-s^2-4\beta)$. Since $\beta^{CAC} > \beta_1^{CAC} = (1-s)s/2$, there exists $q_2^{CAC} > q_1^{CAC}$ for $\beta > \beta_1^{CAC}$, $q_1^{CAC} > q_2^{CAC}$ for $\beta < \beta_1^{CAC}$ and $q_1^{CAC} = q_2^{CAC}$ for $\beta = \beta_1^{CAC}$.

***Proof of Proposition 4***

From Eqs. (A5) and (A6), we obtain $\pi_1^{CAC} - \pi_2^{CAC} = A_3/(4s-s^2-4\beta)^2$, where $A_3 = 4\beta^2 - s(8-5s)\beta + (4-5s+s^2)s^2$. Obviously, Sign $\{\pi_1^{CAC} - \pi_2^{CAC}\}$ = Sign $\{A_3\}$. $A_3$ is a quadratic curve about $\beta \in (0, \beta^{CAC}]$ and $\Delta_3 = 9s^4 > 0$. So, there are two roots for equation $A_3$=0 . One root, $(4-s)s/4 > s/2 = \beta^{CAC}$, always holds. If the other root, $\beta_2^{CAC} = (1-s)s \leq \beta^{CAC}$, holds, we must ensure that $s \leq 0.5$. That is to say, one root, $(4-s)s/4 > \beta^{CAC}$, holds for any $0 < s < 1$ whilst the other root, $\beta_2^{CAC} \leq \beta^{CAC}$, holds for $s \geq 0.5$ and $\beta_2^{CAC} > \beta^{CAC}$ holds for $s < 0.5$.

Since quadratic curve $A_3$ goes upwards, $\pi_1^{CAC} > \pi_2^{CAC}$ always holds for $s \in (0, 0.5)$, but for $s \in [0.5, 1)$, if $0 < \beta < \beta_2^{CAC}$, $\pi_1^{CAC} > \pi_2^{CAC}$ always holds. If $\beta \geq \beta_2^{CAC}$, $\pi_2^{CAC} \geq \pi_1^{CAC}$ always holds.
